# Enzymatic Lipid Oxygenation and Ferroptosis Resistance: A Critical Evaluation of the Lipid Antioxidant Barrier Hypothesis

**DOI:** 10.3390/cimb48100990

**Published:** 2026-09-25

**Authors:** Hao Peng, Chanikarn Srinark, Chunxia Li, Chune Mo, Bihui Li, Joel A. Schick, Minglin Ou

**Affiliations:** 1 Laboratory Center, Guangxi Health Commission Key Laboratory of Glucose and Lipid Metabolism Disorders, The Second Affiliated Hospital of Guilin Medical University, Guilin 541199, China; 2 Genetics and Cellular Engineering Group, Research Unit Signaling and Translation, Helmholtz Zentrum Munich, Ingolstaedter Landstr. 1, 85764 Neuherberg, Germany; 3 Department of Oncology, The Second Affiliated Hospital of Guilin Medical University, Guilin 541199, China; 4 Laboratory Center, Guangxi Key Laboratory of Metabolic Reprogramming and Intelligent Medical Engineering for Chronic Diseases, The Second Affiliated Hospital of Guilin Medical University, Guilin 541199, China

**Keywords:** directed lipid oxygenation, lipoxygenase, lipid antioxidant barrier, conceptual framework, ferroptosis, oxylipins, NRF2, specialized pro-resolving mediators

## Abstract

Lipid peroxidation has long been viewed primarily as a destructive process—a chaotic, free radical-driven cascade culminating in membrane damage and cell death—although its beneficial aspects have been increasingly recognized over the past decades. However, a growing body of evidence challenges this inflexible hypothesis, as exemplified by the recent demonstration that lipoxin A_4_, a product of directed enzymatic lipid oxygenation, actively suppresses ferroptosis. In this review, we synthesize the emerging paradigm of directed lipid oxygenation as a regulated, adaptive mechanism that may function as a physiologically regulated lipid antioxidant barrier. We contrast the stochastic chemistry of non-enzymatic lipid peroxidation with the stereospecific, enzymatically driven oxygenation catalyzed primarily by lipoxygenases (LOXs), cyclooxygenases (PTGSs), and cytochrome P450 enzymes, and hypothesize that these two modes compete for polyunsaturated fatty acid (PUFA) substrates, thereby determining cellular outcomes. We further examine how enzymatic lipid oxygenation generates bioactive oxylipins, including specialized pro-resolving mediators (SPMs), that activate cytoprotective transcriptional programs such as the NRF2 pathway. Central to this framework is the coupled operation of LOX-mediated oxygenation and GPX4-mediated hydroperoxide detoxification: the protective outcome depends on GPX4 as the gatekeeper that channels LOX products toward protective signaling rather than ferroptotic accumulation. The implications for ferroptosis are particularly profound: unchecked lipid peroxidation drives ferroptotic cell death, whereas directed enzymatic lipid oxygenation may oppose it. Whether this constitutes an endogenous barrier remains a hypothesis, and our model does not depend on depletion of the PUFA substrate pool. Finally, we outline therapeutic opportunities and outstanding questions for the field.

## 1. Introduction

The dual character of lipid peroxidation—distinguishing non-enzymatic from enzymatic pathways—has been recognized for over two decades [1]. Building on this established framework, the present review focuses specifically on the implications of this dichotomy for ferroptosis resistance, an angle that has not been systematically explored. The classic narrative of lipid peroxidation in biology is one of damage. Since the foundational work of Halliwell and Gutteridge [2], the field has understood that reactive oxygen species (ROS) initiate a free radical chain reaction in membrane polyunsaturated fatty acids (PUFAs), propagating through lipid peroxyl radical intermediates to generate lipid hydroperoxides (LOOHs). These LOOHs and their breakdown products, reactive aldehydes such as 4-hydroxynonenal (4-HNE) and malondialdehyde (MDA), have been canonically linked to membrane dysfunction, protein crosslinking, DNA damage, and ultimately cell death [2] (Chapter 4: Lipid Peroxidation). This ROS → lipid peroxidation → cell damage cascade has become a cornerstone of oxidative stress biology.

However this narrative, while correct in its essentials, is increasingly recognized as incomplete. Over the past two decades, a substantial body of work has revealed that not all lipid oxygenation events are created equal, and importantly, not all are detrimental. The landmark review by Kuhn et al. [3] argued that mammalian lipoxygenases regarded primarily as pro-inflammatory enzymes are in fact “key players in the regulation of cellular redox homeostasis”. Their analysis challenged the field to move beyond a purely pathological view of lipid oxidation, noting that “although LOXs have been implicated in pathogenesis, their biological roles are not completely understood” [3].

Over the past two decades, a growing body of experimental work has documented conditions under which LOX activity and LOX-derived oxylipins are protective rather than destructive. Biochemically, the coupling between LOX catalysis and peroxide-reducing enzymes was established early: Schnurr et al. showed that phospholipid hydroperoxide glutathione peroxidase (GPX4) controls the activity of 15-lipoxygenase toward complex substrates and preserves the stereospecificity of its oxygenation products, indicating that enzymatic hydroperoxide formation is normally coupled to immediate reduction [4]. Functionally, several classes of LOX-derived products activate cytoprotective programs: electrophilic lipid species activate the NRF2/KEAP1 antioxidant axis [5,6]; 15-HETE and 13-HODE act as endogenous ligands of PPARγ and PPARα [7]; and specialized pro-resolving mediators (SPMs), the terminal products of sequential LOX reactions, orchestrate the resolution of inflammation [8,9]. Most directly relevant to this review, lipoxin A_4_ (LXA_4_), an LOX-derived SPM, was recently demonstrated to suppress ferroptosis in vivo [10]. At the same time, a critical evaluation of this evidence is warranted. First, the canonical electrophilic NRF2 activators that have been characterized experimentally, the nitro-fatty acids, arise from non-enzymatic nitration of unsaturated fatty acids rather than from LOX catalysis, so they demonstrate that lipid oxidation products can signal protection, but not that LOX products specifically do so [5,6]. Second, the same enzymes that generate protective oxylipins can, under different conditions, promote ferroptosis: ALOX15 complexed with PEBP1 generates ferroptotic phospholipid hydroperoxides [11], and ALOX15/15-HpETE aggravates myocardial ischemia–reperfusion injury by promoting cardiomyocyte ferroptosis. Third, ferroptosis can proceed in the absence of LOX activity through non-enzymatic autoxidation [12], indicating that LOX-derived products are neither necessary nor sufficient for ferroptosis execution. The protective versus destructive outcome of enzymatic lipid oxygenation is therefore context-dependent, and this context dependence raises a central chemical question.

The most compelling example that enzymatic lipid oxygenation can protect against ferroptosis is provided by LXA_4_, a trihydroxy eicosanoid generated from arachidonic acid by sequential LOX action (ALOX15 followed by ALOX5, or through transcellular biosynthesis involving platelet ALOX12 and leukocyte ALOX5) [8,13]. Kollareth et al. (2025) demonstrated that LXA_4_ mitigates ferroptosis of alveolar epithelial cells during lung ischemia–reperfusion injury (IRI) [10]. Using hilar-ligation and orthotopic lung transplant models in mice, the authors showed that LXA_4_ administration attenuated pulmonary dysfunction, ferroptosis, and inflammation and reduced the accumulation of oxidized lipids. Notably, this protection was abolished in Fpr2^−/−^ and Nrf2^−/−^ mice, providing genetic evidence that the effect is mediated by LXA_4_ signaling through its receptor FPR2/ALX and strictly requires the NRF2 pathway. Mechanistically, LXA_4_/FPR2 signaling increased NRF2 activation and glutathione concentrations and decreased malondialdehyde (MDA) levels in alveolar type II epithelial cells [10]. The human relevance of this pathway is supported by single-cell transcriptomic evidence of altered ferroptosis gene expression in lung tissue from post-lung-transplant patients [10]. This study is significant for the present framework for three reasons: it demonstrates ferroptosis suppression by a bona fide LOX-derived product in vivo; it identifies a specific receptor (FPR2/ALX) and transcription factor (NRF2) that link a lipid mediator to ferroptosis resistance; and it establishes a plausible therapeutic paradigm. Nevertheless, critical evaluation is required: the protective effect has so far been demonstrated after exogenous administration of the mediator, and confirmation in additional ferroptosis models; the broader SPM field has been subject to methodological scrutiny regarding the quantification of these mediators in biological samples [14,15]; and confirmation in additional ferroptosis models (e.g., erastin- or RSL3-induced ferroptosis) and in other tissues will be needed before the anti-ferroptotic activity of LXA_4_ can be regarded as a general principle.

Why should enzymatically generated hydroperoxides differ from non-enzymatic ones? The protective effects described above are counterintuitive because both LOX catalysis and non-enzymatic peroxidation generate lipid hydroperoxides, the species classically regarded as drivers of oxidative damage. Three physicochemical and biochemical features distinguish enzymatically generated hydroperoxides from their non-enzymatic counterparts, and together they explain how the former can protect rather than promote ferroptosis. (i) HPETEs are chemically distinct from peroxyl radicals. The chain-propagating species of non-enzymatic lipid peroxidation is the lipid peroxyl radical (LOO•), which abstracts bis-allylic hydrogen atoms from neighboring PUFAs and thereby sustains an autocatalytic chain reaction [16]. In contrast, the primary products of LOX catalysis, the hydroperoxyeicosatetraenoic acids (HPETEs) and their phospholipid-esterified counterparts, are closed-shell molecules that lack radical character; they cannot abstract hydrogen atoms and therefore do not propagate chain reactions [12]. This distinction is central to the concept of directed oxygenation: LOX catalysis converts a radical-propagating regime into a non-propagating one, arresting oxidation at the hydroperoxide stage. Critically, however, HPETEs are not intrinsically inert: in the presence of reduced iron (Fe^2+^), they undergo Fenton-like decomposition to alkoxyl radicals that can reinitiate chain peroxidation [12,16]. The protective outcome of enzymatic oxygenation therefore depends on the kinetic competition between hydroperoxide reduction and iron-catalyzed decomposition. (ii) GPX4-dependent rapid reduction. The enzyme that resolves this competition in favor of protection is GPX4 (phospholipid hydroperoxide glutathione peroxidase), which rapidly reduces both free and phospholipid-esterified hydroperoxides, including those generated by LOXs, to the corresponding alcohols [4,17]. Schnurr et al. demonstrated not only that GPX4 reduces 15-LOX-derived hydroperoxides but also that it controls 15-LOX activity on complex substrates and preserves the stereospecificity of its oxygenation products [4]. In this scheme, LOX catalysis and GPX4 operate as a coupled two-step module in which a potentially dangerous intermediate is immediately detoxified. When this coupling fails, as in GPX4-deficient cells that undergo ferroptosis [17,18], LOX-derived hydroperoxides accumulate and become substrates for iron-dependent decomposition, converting a protective module into a ferroptotic one. Consistent with this view, ferroptosis can be executed by non-enzymatic autoxidation when LOX-derived products are not reduced [12]. (iii) Stereospecificity and receptor-mediated signaling. LOX catalysis inserts molecular oxygen at a specific carbon of the PUFA backbone with strict enantioselectivity, generating defined products such as 15S-HpETE and 12S-HpETE [19,20], whereas non-enzymatic peroxidation yields complex mixtures of positional isomers as racemates [21]. This stereochemical distinction has functional consequences. First, defined enantiomers are recognized by dedicated signaling machinery: FPR2/ALX binds LXA_4_ stereoselectively and couples to NRF2 activation [20,22], whereas racemic oxidation products are not agonists of these receptors and instead serve primarily as biomarkers of oxidative damage. Second, cells handle stereospecific products through defined metabolic routes, including the class-specific incorporation of HETE isomers into membrane phospholipids, allowing controlled membrane remodeling rather than indiscriminate damage. Enzymatic oxygenation is thus not merely a cleaner form of oxidation; it generates defined molecular signals that cells can interpret, while non-enzymatic peroxidation generates chemical noise. Taken together, these considerations specify the boundary conditions under which enzymatic lipid oxygenation protects rather than damages: the generated hydroperoxides must be rapidly reduced by GPX4, must be produced in a stereospecific manner that permits receptor-mediated signaling, and must not exceed the cellular peroxide-reducing capacity, whose interplay with LOX activity was recognized early [1].

Ferroptosis is an iron-dependent form of regulated cell death driven by the uncontrolled peroxidation of membrane PUFAs [23,24]. The evidence summarized above shows that the enzymatic generation of lipid hydroperoxides can, under defined conditions, oppose this process. The central question that this review seeks to address is: under what conditions do enzymatically generated lipid hydroperoxides protect against, rather than promote, ferroptosis?

While the recognition that LOXs can be protective represents important progress, the mechanistic basis of this protection has not been fully integrated. Several previous reviews have explored aspects of the relationship between lipid oxidation and ferroptosis [23,25]. The present review builds on these foundations by specifically examining how the dichotomy between enzymatic and non-enzymatic lipid oxygenation can be applied to understand ferroptosis resistance mechanisms.

If lipid oxygenation can be both stochastic (free radical-driven) and directed (enzyme-catalyzed), then the biological outcome must depend not merely on the extent of oxidation, but on its mode. We hypothesize that directed enzymatic lipid oxygenation may represent an evolutionarily conserved strategy for constructing a lipid antioxidant barrier: a controlled, physiologically regulated mechanism that consumes oxidizable substrates, generates protective signaling molecules, and activates cellular defense programs.

We first contrast the chemistry of directed versus non-enzymatic lipid oxygenation (Section 2), then synthesize the three mechanisms underlying the lipid barrier model (Section 3), examine the implications for ferroptosis (Section 4), and finally discuss therapeutic opportunities and future perspectives (Section 5). In this review, we use the term ‘enzymatic lipid oxygenation’ to refer to enzyme-catalyzed, stereospecific lipid oxygenation and ‘non-enzymatic lipid peroxidation’ to refer to free radical-driven chain peroxidation. The term ‘directed’ is used sparingly for stylistic variation.

In this review, we use the following conventions: statements without qualifiers represent experimentally established mechanisms; statements preceded by “we hypothesize” or “we propose” represent the authors’ interpretation of the available evidence; and “this model predicts” introduces testable but untested predictions. Speculative components are explicitly labeled as such.

## 2. Directed Versus Non-Enzymatic Lipid Peroxidation

### 2.1. Non-Directed Lipid Oxidation: The Free Radical Chain Reaction Paradigm

Non-enzymatic lipid peroxidation proceeds through a well-characterized free radical chain reaction comprising three phases: initiation, propagation, and termination [2]. In the initiation phase, a radical species (typically hydroxyl radical, •OH, or alkoxyl radical, RO•) abstracts a hydrogen atom from a bis-allylic methylene group of a PUFA, generating a carbon-centered lipid radical (L•). The carbon-centered lipid radical (L•) is resonance-stabilized through double-bond rearrangement, yielding a conjugated diene radical; reaction with O2 at the terminal or internal positions of this conjugated system yields conjugated lipid peroxyl radicals (LOO•) [26]. Lipid alkoxyl radicals (LO•) and hydroxyl radicals (•OH), both originating from the decomposition of lipid hydroperoxides, also contribute to chain propagation [27]. The propagation phase ensues when LOO• abstracts hydrogen from an adjacent PUFA, perpetuating the chain reaction. Termination occurs when two radical species combine to form non-radical products.

This process is fundamentally non-discriminating as it does not distinguish between specific PUFAs, specific positions within the acyl chain, or specific stereochemical configurations. The products are complex racemic mixtures of positional and geometric isomers. Iron, particularly through Fenton chemistry (Fe^2+^ + H_2_O_2_ → Fe^3+^ + •OH + OH^−^), is a critical catalyst that amplifies initiation [2]. The biological consequence of uncontrolled chain peroxidation is catastrophic: membrane barrier function collapses, and reactive aldehydes modify proteins and DNA, triggering inflammatory responses and cell death.

The free radical chain reaction is illustrated schematically in Figure 1. It should be noted that a low level of non-enzymatic lipid peroxidation is essential for normal cellular redox signaling, and that complete suppression can lead to reductive stress.

### 2.2. Directed Lipid Oxygenation: The Enzymatic Paradigm

In stark contrast, enzymatic lipid oxygenation is a site-specific, stereoselective process. Lipoxygenases (LOXs) constitute the principal family of enzymes catalyzing directed lipid oxygenation. These non-heme iron-containing dioxygenases insert molecular oxygen into specific positions of PUFA substrates with exquisite regio- and stereochemical control [3,28]. Mammalian LOXs are classified according to their positional specificity of oxygenation on arachidonic acid: ALOX5, ALOX12, and ALOX15, each generating the corresponding hydroperoxyeicosatetraenoic acid (HPETE).

While LOXs are the primary focus of this review due to their direct action on membrane-esterified PUFAs, it should be noted that other enzyme families, including cyclooxygenases (PTGSs) and cytochrome P450 (CYP) monooxygenases, also catalyze stereospecific lipid oxygenation. PTGSs generate prostaglandin G_2_/H_2_ intermediates [29], while CYP monooxygenases generate epoxy fatty acids (EETs) as well as ω-hydroxylated and ω-1 hydroxylated PUFAs [22,30]. Unlike ALOX and PTGS products, CYP-derived products are chemically stable and do not initiate secondary lipid peroxidation or radical chain reactions. These enzyme systems, though distinct in mechanism and product profiles, share the key feature of directed, site-specific oxygenation that distinguishes them from non-enzymatic peroxidation. The principles of the lipid barrier model may therefore extend beyond LOXs, though LOXs occupy a privileged position due to their direct access to membrane-embedded substrates.

The structural basis for LOX specificity lies in the depth of the substrate-binding pocket and the orientation of the substrate within the enzyme active site. Brash (1999) provided a comprehensive analysis of LOX catalysis, highlighting the “arachidonic acid cassette” model in which the fatty acid is bound with its methyl end buried in a hydrophobic pocket, exposing specific carbon atoms to the active-site iron [19]. The N-terminal β-barrel domain of mammalian LOXs has been shown to be important for membrane association, though it is not essential for catalytic activity, suggesting a regulatory role in substrate access [31].

The oxygenation of membrane phospholipids by ALOX enzymes was first described by Schewe et al. (1975) in the context of reticulocyte maturation; subsequent work by Brash et al. (1987) characterized the ability of soybean ALOX to oxygenate phospholipid-esterified fatty acids in detergent-containing systems [32,33]. This ability to act directly on membrane-embedded substrates distinguishes LOX-mediated oxygenation from many other lipid-modifying enzymes and positions LOXs as direct participants in membrane lipid remodeling. It is important to note that the capacity to oxygenate complex esterified substrates is not uniform across ALOX isoforms. ALOX15 and ALOX15B are capable of directly oxygenating phospholipids, whereas ALOX12, ALOX5, ALOX12B, and ALOXE3 appear to preferentially oxygenate free fatty acids.

The product profiles of directed and non-enzymatic lipid oxygenation are diagnostically distinct. Enzymatic lipid oxygenation generates a single major enantiomer (typically S-configuration) at a specific carbon position, while non-directed peroxidation yields racemic mixtures of multiple positional isomers. These differences are analytically resolvable by chiral chromatography and mass spectrometry, as reviewed by Ivanov et al. [20].

The evolutionary distribution of LOXs which is present throughout the plant and animal kingdoms, as reviewed by Kuhn and Thiele (1999) [28] underscores their fundamental biological importance. Plants utilize LOX products as signaling molecules in wound response, pathogen defense, and development, suggesting an ancient origin for the protective functions of enzymatic lipid oxygenation.

The enzymatic oxygenation pathway is summarized in Figure 1.

### 2.3. Competition for PUFA Substrates

The simultaneous presence of enzymatic and non-enzymatic oxygenation pathways creates a fundamental competition for a shared substrate pool. Membrane PUFAs, particularly arachidonic acid (20:4), linoleic acid (18:2), and docosahexaenoic acid (22:6), represent a finite substrate pool at any given moment that can be consumed by either pathway, although continuous lipid remodeling and fatty acid turnover can replenish this pool [34].

This competition is shaped by several molecular determinants. First, the subcellular localization of PUFA pools matters: LOXs preferentially oxygenate esterified PUFAs within membrane phospholipids [32], while free radical species attack both free and esterified PUFAs indiscriminately. Second, the physical proximity of LOX enzymes to their substrates further biases competition toward enzymatic oxygenation: certain LOX isoforms possess membrane-binding domains that provide a “first-mover advantage” [31]. Third, the four factors governing the balance between these pathways, LOX expression and activity, catalytic iron availability, GPX4 capacity, and reduced glutathione (GSH) levels, collectively determine whether directed or non-enzymatic lipid oxygenation predominates.

These four factors, namely LOX activity, catalytic iron availability, GPX4 capacity, and GSH levels, will re-emerge throughout this review as the key determinants of whether the lipid barrier is maintained or breached (see Section 3 and Section 4).

It is important to recognize that the primary products of ALOX catalysis (HPETEs) are themselves hydroperoxides and, if not rapidly reduced, can undergo iron-catalyzed decomposition to radicals that initiate secondary peroxidation. Thus, the protective outcome of enzymatic oxygenation depends critically on the efficiency of the cellular peroxide-reducing capacity (primarily GPX4), not merely on the mode of initial oxygenation [17].

## 3. The Lipid Antioxidant Barrier Model

The concept of a “lipid antioxidant barrier” represents an integrative framework for understanding how directed enzymatic lipid oxygenation may be protective rather than destructive. We hypothesize that this barrier operates through three interconnected mechanisms: substrate sequestration, formation of protective oxidation end-products, and activation of antioxidant transcriptional programs.

### 3.1. Substrate Sequestration: A Hypothesis, Not an Established Mechanism

One proposed mechanism of enzymatic lipid oxygenation is substrate sequestration: by consuming PUFAs through controlled enzymatic reactions, LOXs effectively deplete the substrate pool available for chaotic free radical chain peroxidation. The notion that LOXs participate in maintaining redox homeostasis as articulated by Kuhn et al. [3] provides the conceptual foundation for this mechanism. In this model, LOX-mediated oxygenation is hypothesized to function as a physiological “sink” for PUFAs, converting them from potentially dangerous chain reaction substrates into stable, biologically useful products.

This is conceptually analogous to a “controlled burn” in fire ecology, where intentional, managed combustion removes fuel and thereby may prevent catastrophic wildfires. While direct quantitative evidence for substrate sequestration in mammalian systems remains limited, the concept is supported by studies in plant systems where LOX-mediated oxylipin production has been directly linked to reduced non-enzymatic lipid peroxidation during stress responses [35,36]. This conservation is consistent with an ancestral or plant-specific role for substrate sequestration, but it does not establish a contribution to barrier function in mammalian membranes.

We wish to emphasize that this analogy is intended as an illustrative heuristic, not as mechanistic evidence. The quantitative differences between the biological system (nanomolar concentrations, membrane-embedded enzymes) and macroscopic fire ecology limit the direct applicability of this comparison.

More fundamentally, it must be acknowledged that direct experimental evidence that enzymatic oxygenation meaningfully competes with free-radical lipid peroxidation for PUFA substrates in vivo remains limited. Key unresolved questions include: (i) Are LOX-catalyzed reaction rates sufficient to outcompete free radical propagation at the membrane? (ii) Does competition occur before radical chain reactions become self-sustaining? (iii) Is PUFA availability genuinely rate-limiting under physiological conditions? These questions require focused experimental investigation.

It should be noted that the substrate sequestration hypothesis faces quantitative challenges. Under physiological conditions, the OH-PUFA/PUFA ratio in membrane lipids is typically 0.01–0.1%, and even under conditions of extreme ALOX overexpression (as in rabbit reticulocytes) this ratio does not exceed 5% [1]. This suggests that PUFA depletion is quantitatively insufficient to be a mechanism of barrier function in mammalian cells. We therefore do not include substrate sequestration in the model presented in Section 3.5; the model does not depend on it, and the mechanism is retained only as a hypothesis with a possible ancestral or plant-specific role.

An additional consideration is that extensive oxygenation of membrane PUFAs may alter membrane fluidity and biophysical properties, potentially impairing membrane function [37]. These potential trade-offs require further investigation.

### 3.2. Formation of Protective Oxidation End-Products

The products of enzymatic lipid oxygenation, collectively termed [oxylipins], differ from the breakdown products of non-enzymatic peroxidation in their defined stereochemistry and in their receptor-mediated biological activities; this difference does not by itself establish that enzymatic oxygenation is protective.

Stability: HPETEs are the initial products of LOX catalysis and are themselves hydroperoxides, like the peroxyl radical intermediates (LOO•) of non-enzymatic peroxidation; both must be reduced to be detoxified. HPETEs are rapidly reduced by cellular peroxidases (including GPX4) to the corresponding HETEs, which are chemically stable end-products that can be incorporated into membrane phospholipids or metabolized to more complex signaling molecules [16,38]. The distinction that matters for protection is therefore not that enzymatic products lack radical character, but that stereospecific oxygenation generates defined products which act on defined receptors, whereas the products of non-enzymatic peroxidation are racemic and have no defined receptor targets. Whether this distinction translates into protection depends on the capacity of the reduction machinery (Section 3.5).

Signaling Functions: Among the most biologically significant products of enzymatic lipid oxygenation are the specialized pro-resolving mediators (SPMs), a family of lipid mediators including lipoxins (derived from arachidonic acid, AA), resolvins (from EPA and DHA), protectins (from DHA), and maresins (from DHA). The pioneering work of Serhan (2014) established that these molecules are not merely inert metabolites but potent agonists of resolution physiology, actively promoting the termination of inflammation and the restoration of tissue homeostasis [8]. SPMs signal through specific G protein-coupled receptors (e.g., FPR2/ALX for lipoxin A_4_, GPR32 for resolvin D1) to coordinate macrophage-mediated clearance of apoptotic cells, neutrophil recruitment cessation, and tissue repair. The SPM network has expanded substantially, with recent updates identifying additional receptor pairs and tissue-specific expression patterns [9]. The generation of SPMs requires sequential actions of different LOX isoforms. For example, lipoxin A_4_ is generated through the sequential actions of ALOX15 and ALOX5 or through transcellular biosynthesis involving platelet ALOX12 and leukocyte ALOX5 [8,13]. This multi-enzyme requirement underscores the highly regulated nature of enzymatic lipid oxygenation.

The SPM concept has recently been critically evaluated. Schebb et al. questioned the robustness of the biosynthetic pathways and analytical methods underlying SPM identification, while O’Donnell et al. questioned the detection and quantitation of SPMs in biological samples [14,15]. These critiques highlight the need for rigorous analytical standards in SPM research. We acknowledge these limitations. In the ferroptosis studies reported to date, protection was produced by exogenous administration of LXA4 rather than by an increase in endogenous mediator production, and endogenous tissue concentrations of LXA4 are frequently at or below validated limits of detection. The protective arm of the model therefore cannot be assumed to operate in tissues where FPR2 or comparable SPM receptors are expressed at low or undetectable levels.

Heterogeneity of oxylipin functions. Not all oxylipins are electrophilic or activate KEAP1/NRF2, and it is important to specify which classes of LOX-derived products carry protective signaling capacity (Table 1). Electrophilic lipids that modify KEAP1 cysteines—such as nitro-fatty acids [5,6] and certain cyclopentenone eicosanoids—constitute a chemically distinct subclass; most LOX-derived products (HETEs, HPETEs, and their phospholipid-esterified forms) lack the alpha,beta-unsaturated carbonyl electrophile required for efficient KEAP1 modification and instead act through dedicated receptors (FPR2/ALX for LXA4 [10]; GPR32 for resolvin D1 [9]) or nuclear receptors (PPARgamma for 15-HETE and 13-HODE [7]). HPETEs themselves are not receptor agonists and function primarily as metabolic intermediates. The protective actions of enzymatic lipid oxygenation are therefore mediated by defined product classes acting on defined targets, and the assumption that all LOX products are protective should be explicitly avoided.

### 3.3. Activation of Antioxidant Circuits

Evidence for the protective function of enzymatic lipid oxygenation comes from the demonstration that defined oxylipin products activate cellular signaling through dedicated receptors (Section 3.2), whereas NRF2-dependent antioxidant programs are activated by electrophilic lipids, which are largely of non-enzymatic origin.

NRF2 Activation by Electrophilic Lipids: The transcription factor NRF2 is the master regulator of the antioxidant response, controlling the expression of over 200 genes including heme oxygenase-1 (HO-1), NAD(P)H:quinone oxidoreductase 1 (NQO1), and glutathione S-transferases. Under basal conditions, NRF2 is targeted for proteasomal degradation by its inhibitor KEAP1. Electrophilic modification of KEAP1 at specific cysteine residues (particularly Cys151, Cys273, and Cys288) disrupts this interaction, leading to NRF2 stabilization and nuclear translocation. Structural studies have revealed that the ETGE and DLG motifs of NRF2 act as a hinge and latch to regulate KEAP1 binding [39].

Kansanen et al. (2011) demonstrated that electrophilic nitro-fatty acids, which arise from non-enzymatic nitration of unsaturated fatty acids rather than from LOX catalysis, such as oxidation products of unsaturated fatty acids activate NRF2 through a mechanism involving KEAP1 cysteine modification [5]. This work provided direct evidence that the products of lipid oxidation can function as signaling molecules that upregulate the cell’s own antioxidant capacity. Building on this concept, Zheng et al. (2014) showed that the electrophilic nitrofatty acids 9- and 10-nitrooleic acid induce the expression of stress proteins and antioxidants in keratinocytes, including HO-1, heat shock proteins, and thioredoxin reductase [6]. These studies establish that products of non-enzymatic lipid oxidation can activate NRF2, but they do not show that LOX-derived products do so; the enzymatic arm of the model reaches NRF2 only indirectly, through FPR2-mediated signaling [10], a weaker and receptor-restricted route.

Beyond NRF2, certain oxylipins also activate peroxisome proliferator-activated receptors (PPARs), particularly PPARγ and PPARα. For example, 15-HETE and 13-HODE are endogenous PPARγ ligands that can modulate lipid metabolism and inflammatory gene expression [7]. This represents a parallel pathway through which enzymatic lipid oxygenation products influence cellular redox and metabolic states, complementing the NRF2-centered transcriptional response. It should be noted that the activation of PPAR isoforms by ALOX products remains incompletely characterized. Only selected HODE and HETE isomers have been tested, and the enantioselectivity of PPAR activation has not been systematically explored. Thus, the physiological relevance of this pathway requires further validation [7].

#### GPX4 and the Selenium-Containing Defense: A Bidirectional Relationship

Glutathione peroxidase 4 (GPX4) is the primary enzyme responsible for reducing phospholipid hydroperoxides to the corresponding alcohols, thereby detoxifying both enzymatic and non-enzymatic lipid peroxidation products. Yang et al. (2014) established GPX4 as the central gatekeeper of ferroptosis, demonstrating that GPX4 loss leads to lethal lipid peroxidation that can be suppressed by iron chelators and lipophilic antioxidants [18]. The regulatory roles of GPX4 in ferroptosis have been further elaborated, revealing its function as a critical node integrating selenium metabolism, lipid peroxidation, and cell death signaling [17]. The connection to enzymatic lipid oxygenation is bidirectional: GPX4 is capable of reducing phospholipid hydroperoxides generated by ALOX enzymes in vitro [4,10]. and it is widely assumed that this activity extends to ALOX-derived HPETEs in vivo, although direct experimental confirmation in cellular systems remains limited, while the signaling products of enzymatic lipid oxygenation (such as lipoxin A_4_) can enhance GPX4 expression through NRF2-dependent mechanisms [4].

### 3.4. Evolutionary Conservation of the Lipid Barrier Concept

The evolutionary distribution of LOXs provides compelling circumstantial evidence for the protective functions of enzymatic lipid oxygenation. LOXs are present throughout the plant and animal kingdoms [28], and genome-wide analyses have revealed that LOX gene families have undergone significant expansion and diversification across angiosperms, suggesting that the capacity for directed lipid oxygenation has been selectively maintained over hundreds of millions of years [40]. ALOX isoforms have also been detected in a small fraction of bacterial species (~0.1–1% of sequenced genomes). Phylogenetic analyses suggest that bacterial ALOX genes were acquired via horizontal gene transfer from eukaryotic organisms, and no ALOX genes have been identified in archaea [20,41].

In plants, LOX-derived oxylipins function primarily as signaling molecules in wound response, pathogen defense, and developmental programs [35,36]. Notably, plants lack adaptive immune systems and rely on these rapid, localized lipid signals to coordinate stress responses, a context in which the protective, rather than pro-inflammatory, functions of LOXs are most apparent. The jasmonate pathway, for example, uses LOX-derived products (specifically 13-hydroperoxy linolenic acid) to activate defense gene expression and limit oxidative damage during biotic stress [42]. This pathway represents one of the most well-characterized examples of a lipid antioxidant barrier in any organism: controlled LOX-mediated oxygenation generates signaling molecules that preemptively strengthen cellular defenses.

In plants, LOX activity has a dual role: it generates signaling molecules (jasmonates) that coordinate defense responses, but uncontrolled LOX activity can promote lipid oxidation and rancidity, as seen in vegetable oils and stored plant products. The protective role of plant LOX activity is therefore context-dependent and requires tight regulation, mirroring the situation in animals.

In animals, LOXs have evolved additional complexity, including the capacity for transcellular biosynthesis of SPMs and integration with the immune system. Yet the ancestral protective function of consuming oxidizable substrates and generating signaling molecules that coordinate cellular defense appears to have been retained. The emergence of ferroptosis as a regulated cell death pathway in vertebrates may have co-opted LOX enzymes that originally served protective roles, creating a “double-edged sword” in which the same enzymatic machinery can either preserve or destroy membrane integrity depending on context [25].

The broad evolutionary conservation of LOX enzymes across plants, animals, and some bacteria [28,40,41] suggests fundamental biological importance, although the nature of this importance—whether antioxidant, signaling, or metabolic—remains to be determined. We speculate that the ancestral function may have been related to membrane remodeling or signaling, with antioxidant functions representing a later specialization in some lineages. The evolutionary conservation of LOXs therefore provides circumstantial, rather than direct, support for the barrier model.

### 3.5. The Lipid Barrier Model: An Integrative Framework

Synthesizing the above mechanisms, we hypothesize the Lipid Barrier Model as a conceptual framework for understanding enzymatic lipid oxygenation as a protective process.

The term “barrier” is used here in two complementary senses. Physically, it refers to the membrane lipid environment in which PUFAs are oxygenated by enzymes in a stereospecific and enzymatically controlled manner. Conceptually, it refers to the integrated system of enzymes (LOXs), signaling molecules (oxylipins/SPMs), and transcriptional programs (NRF2) that sustain this state. The lipid antioxidant barrier is therefore best described as a process rather than as a pre-existing state of the membrane, and it does not require that PUFAs be oxidized or depleted.

LOX catalytic activity is not protective per se. It is protective only when the coupled reduction machinery (GPX4, GSH, CoQ/FSP1) is sufficient and substrate access is not redirected to phospholipid substrates; when reduction fails, the same enzymes contribute to ferroptotic execution. On that basis, the barrier comprises three components: (1) an oxygenation component of LOX isoforms whose contribution is the stereospecific generation of defined products (HETEs and SPMs) that act through dedicated receptors, rather than the consumption of PUFA substrate (Figure 2, upper pathway); (2) a signaling component of oxylipins including HETEs, SPMs, and electrophilic lipids that signal to neighboring cells and activate transcriptional programs (Figure 2, upper pathway); and (3) a transcriptional component made of NRF2-driven upregulation of antioxidant enzymes including HO-1, GPX4, and glutathione synthesis enzymes, creating a sustained state of heightened resistance (Figure 2, upper pathway). The model does not depend on depletion of the substrate pool (Section 3.1).

The barrier is dynamic. Its composition and activity can be modulated in response to cellular state, tissue context, and environmental challenges. When functioning properly, it may prevent the transition from controlled, protective oxygenation to uncontrolled, destructive peroxidation. However, when the barrier collapses, either through iron overload, GPX4 loss, or overwhelming oxidative challenge, the same LOX enzymes that maintain the barrier can instead fuel ferroptotic death (Figure 2, lower pathway). Failure of the barrier may be a prerequisite for ferroptosis.

## 4. Implications for Ferroptosis

### 4.1. Current Ferroptosis Paradigm

Ferroptosis, first defined by Dixon et al. (2012) as an iron-dependent form of non-apoptotic cell death, is now recognized as a central mechanism in diverse pathological contexts including neurodegeneration, ischemia–reperfusion injury, and cancer therapy [24]. The canonical ferroptosis pathway is characterized by three essential features: iron accumulation, glutathione depletion, and lipid peroxidation. As reviewed by Gao et al. (2025), “ferroptosis is characterized by iron accumulation and increased lipid peroxidation” [43]. The field has matured substantially, with the tenth anniversary of its discovery marked by comprehensive reviews that emphasize the diversity of ferroptosis mechanisms, its physiological roles, and emerging therapeutic applications [23].

The current molecular understanding positions GPX4 and the cystine/glutamate antiporter system Xc^−^ (SLC7A11/SLC3A2) as the central regulators. Inhibition of system Xc^−^ (e.g., by erastin) depletes intracellular cysteine, limiting glutathione synthesis and compromising GPX4 activity. Direct GPX4 inhibition (e.g., by RSL3) bypasses the cysteine requirement. In either case, the consequence is the accumulation of phospholipid hydroperoxides to lethal levels, culminating in membrane rupture and cell death [18,24].

This canonical model implicitly assumes that all lipid oxygenation is equivalent but is challenged by the lipid barrier model. If enzymatic oxygenation generates protective products and consumes pro-ferroptotic substrates, then the balance between directed and non-enzymatic lipid oxygenation, rather than the absolute extent of lipid oxidation, may be the critical determinant of ferroptosis sensitivity.

### 4.2. The Ferroptosis Regulatory Network Beyond GPX4

The canonical GPX4-centered view of ferroptosis has been substantially extended by the discovery of parallel, GPX4-independent antioxidant systems, each of which intersects with the lipid barrier model.

ACSL4 and LPCAT3 define the substrate supply. Acyl-CoA synthetase long-chain family member 4 (ACSL4) preferentially activates arachidonoyl (20:4) and adrenoyl (22:4) CoA esters, and lysophosphatidylcholine acyltransferase 3 (LPCAT3) esterifies them into membrane phospholipids [44,45]. Cells lacking ACSL4 are profoundly resistant to ferroptosis, indicating that PUFA incorporation—the very substrate pool on which both directed and non-enzymatic oxygenation act—is a decisive upstream determinant of ferroptosis sensitivity. In the lipid barrier framework, ACSL4/LPCAT3 determine the total oxidizable substrate, whereas the LOX/GPX4 module determines the mode of its oxygenation.

FSP1/CoQ. Ferroptosis suppressor protein 1 (FSP1, formerly AIFM2) operates in parallel to GPX4 by regenerating ubiquinol (CoQH2), a lipophilic radical-trapping antioxidant, at membranes [46,47]. FSP1 thereby suppresses the propagation phase of lipid peroxidation regardless of its mode of initiation, providing a second layer of defense independent of both LOX activity and GSH.

DHODH. Dihydroorotate dehydrogenase (DHODH) in the mitochondrial inner membrane mediates a ubiquinone-dependent ferroptosis defense that parallels GPX4 in the mitochondrial compartment; DHODH inhibition sensitizes GPX4-low tumors to ferroptosis.

GCH1/BH4. GTP cyclohydrolase 1 (GCH1) and its product tetrahydrobiopterin (BH4) confer ferroptosis resistance through CoQ synthesis and the direct radical-trapping activity of BH4, accompanied by remodeling of membrane phospholipid composition [48,49].

Collectively, these systems define a ferroptosis regulatory network in which the lipid barrier model occupies a defined position: LOX-mediated oxygenation and GPX4-dependent reduction constitute one coupled module, operating alongside ACSL4/LPCAT3-dependent substrate supply, FSP1/CoQ- and DHODH-mediated radical interception, and GCH1/BH4-dependent lipid remodeling. An integrated view of ferroptosis resistance must therefore consider the state of the entire network rather than GPX4 alone.

### 4.3. Pro-Ferroptotic LOX Actions

A balanced assessment of the lipid barrier model requires acknowledging that LOX enzymes are not inherently protective or destructive. Their biological outcomes depend critically on cellular context. Several LOX isoforms have been directly implicated in ferroptosis execution, and these pro-ferroptotic roles must be carefully integrated into any unifying framework.

ALOX15 and the PEBP1 Scaffold: The most well-characterized pro-ferroptotic LOX mechanism involves ALOX15 and its interaction with PEBP1 (phosphatidylethanolamine-binding protein 1). Zhao et al. (2020) demonstrated that PEBP1 acts as a scaffold that redirects ALOX15 from free fatty acid substrates to phospholipid-embedded PUFAs, specifically phosphatidylethanolamine (PE) species [50]. This redirected activity generates 15-hydroperoxy-PE species that serve as ferroptotic death signals. The interaction between ALOX15 and PEBP1 at the membrane surface has been further characterized by molecular dynamics simulations and biochemical studies, revealing specific membrane regions that facilitate the formation of the pro-ferroptotic complex [51]. The identification of selective inhibitors of the ALOX15/PEBP1 complex that do not interfere with ALOX15 biosynthesis provides additional validation of this mechanism and suggests potential therapeutic strategies for ferroptosis-associated pathologies [52]. It should be noted that ALOX15 possesses intrinsic capacity to oxygenate membrane phospholipids independently of adaptor proteins such as PEBP1. Whether this PEBP1-independent activity contributes to ferroptosis signaling or to barrier maintenance remains to be determined [4].

In vivo evidence for the involvement of ALOX15 in ferroptosis is emerging. In a mouse model of myocardial ischemia–reperfusion injury, cardiomyocyte-specific deletion of Alox15 alleviated injury and restored cardiac function, whereas 15-HpETE, the primary Alox15 product, triggered cardiomyocyte ferroptosis [11]. These findings indicate that ALOX15 can act as a pro-ferroptotic effector in vivo, at least in the context of ischemia–reperfusion. Regarding the ALOX15/PEBP1 axis, PEBP1 (RKIP)-deficient mice have been generated and characterized in the context of prostate cancer [53], providing a tool for future investigation of the ALOX15/PEBP1 complex in ferroptosis in vivo; however, such studies have not yet been reported, and the ferroptosis phenotype of PEBP1-/- mice remains to be determined.

ALOX12: ALOX12 has also been linked to ferroptosis in multiple contexts. Chu et al. (2019) demonstrated that ALOX12 is required for p53-mediated tumor suppression through a distinct ferroptosis pathway that is independent of the canonical SLC7A11/GPX4 axis [54]. In this context, p53 represses SLC7A11, which releases the inhibitory effect on ALOX12 and thereby promotes ALOX12-dependent ferroptotic cell death. The p53-ALOX12 axis therefore places ALOX12 among the effectors of p53-dependent ferroptosis rather than among its transcriptional targets. ALOX12 activation has also been implicated in ferroptosis during toxicant-induced injury [54]. To date, in vivo evidence for ALOX12 involvement in ferroptosis in knockout mouse models remains limited, and this represents an important area for future investigation.

The role of ALOX5 in ferroptosis remains controversial. While some studies have implicated ALOX5 in ferroptosis in melanoma cells, where ALOX5 promotes autophagy-dependent ferroptosis through the AMPK/mTOR pathway [55], other reports suggest that ALOX5 inhibition does not protect against ferroptosis, as the cytoprotective effects of most LOX inhibitors, including isoform-selective compounds, have been attributed to their radical-trapping antioxidant activity rather than specific enzyme inhibition [12]. ALOX5-dependent ferroptosis has since been demonstrated in a Huntington’s disease model, in which ALOX5 deletion was protective [56]. Whether ALOX5 acts directly on phospholipid substrates or indirectly through leukotriene signaling remains unresolved.

Determinants of Pro- versus Anti-ferroptotic LOX Outcomes: The transition from protective to detrimental LOX activity is governed by several interacting factors: (1) LOX isoforms, ALOX15, ALOX12, and ALOX5 have distinct substrate preferences and product profiles that dictate their biological outcomes; (2) substrate context, LOX oxygenation of free fatty acids generates signaling molecules (HETEs, SPMs), while oxygenation of phospholipid-esterified PUFAs may generate ferroptotic signals, particularly when directed by scaffolding proteins such as PEBP1 [50,51]; (3) GPX4 capacity, when GPX4 is functional, LOX-derived HPETEs are detoxified to stable HETEs; when GPX4 is compromised, the same LOX products accumulate as phospholipid hydroperoxides and become lethal [57,58]; (4) redox tone, mild oxidative stress may activate protective LOX/NRF2 circuits, whereas severe oxidative stress overwhelms the barrier and converts LOX activity to a death-execution function.

This dual nature does not invalidate the lipid barrier concept but rather refines it. When reduction capacity is adequate and iron is low, LOX activity is tolerated without net harm and its products contribute to protection; the generation of those products, rather than the consumption of substrate, is the contribution of LOX catalysis. When the barrier fails (GPX4 loss, iron overload, PEBP1-mediated substrate redirection), the same enzymatic machinery can execute cell death. The lipid barrier model thus accommodates, rather than ignores, the pro-ferroptotic roles of LOXs. The precise roles of individual ALOX isoforms in ferroptosis remain incompletely defined, and systematic comparisons across isoforms using standardized ferroptosis assays are needed.

### 4.4. Evidence Inconsistent with or Modifying the Lipid Barrier Model

Several lines of evidence appear inconsistent with, or substantially modify, the lipid barrier model, and a balanced assessment requires that they be stated explicitly.

First, pharmacological LOX inhibition has been reported to protect against ferroptosis in multiple experimental systems [59,60], observations that are difficult to reconcile with the view that LOX activity is predominantly barrier-maintaining. It should be noted, however, that many LOX inhibitors also act as radical-trapping antioxidants, and their cytoprotective effects can occur independently of enzyme inhibition [12]; isoform-selective genetic approaches are therefore required to interpret these findings.

Second, LOX products contribute directly to pathology in defined disease models: ALOX15/15-HpETE aggravates myocardial ischemia–reperfusion injury by promoting cardiomyocyte ferroptosis [11], and ALOX15/PEBP1-generated oxygenated phosphatidylethanolamines serve as ferroptotic death signals [50,61].

Third, enzymatic oxygenation itself generates phospholipid hydroperoxides; when the coupled reduction by GPX4 fails—through selenium deficiency, GPX4 inhibition, or overwhelming substrate load—LOX-derived hydroperoxides accumulate and feed iron-dependent decomposition, converting the same enzymatic module into a ferroptosis-promoting one [4,12,17].

These observations define the “double-edged sword” character of LOX activity: whether enzymatic oxygenation protects or kills depends on the integrity of the coupled reduction machinery, the substrate context (free fatty acid versus phospholipid-esterified), and iron availability. The lipid barrier model does not deny these findings; rather, it specifies the conditions under which LOX activity is protective and the conditions under which it is not.

### 4.5. Unsolved Questions Regarding ALOX Isoform Specificity in Ferroptosis

Despite substantial progress in linking ALOX isoforms to ferroptosis, the precise roles of individual family members remain incompletely defined, and several critical questions persist. The following paragraphs summarize the current state of evidence and highlight key knowledge gaps for each major ALOX isoform implicated in ferroptosis biology.

ALOX15 (15-Lipoxygenase) and the PEBP1 Axis

The ALOX15/PEBP1 interaction represents the most mechanistically characterized pro-ferroptotic ALOX pathway. Zhao et al. demonstrated that PEBP1 acts as a scaffold that redirects ALOX15 from free fatty acid substrates toward phosphatidylethanolamine (PE) species in membranes, generating 15-hydroperoxy-PE that serve as ferroptotic death signals [50]. Molecular dynamics simulations have further revealed specific membrane regions that facilitate this pro-ferroptotic complex formation [51] and selective inhibitors of the 15LOX/PEBP1 complex that do not interfere with ALOX15 biosynthesis have been identified [52].

The fundamental distinction between free fatty acid oxygenation and PE-esterified fatty acid oxygenation lies in (i) the product’s integration into the membrane bilayer, where PE hydroperoxides disrupt membrane packing and promote pore formation; (ii) the inability of PE-esterified ALOX products to be rapidly metabolized and released as free HETEs, so that their detoxification requires phospholipid hydroperoxide-reducing enzymes such as GPX4; and (iii) the specific interaction of oxidized PE species with ferroptosis execution machinery [61].

However, several limitations deserve emphasis. First, it remains unclear whether the ALOX15/PEBP1 axis is essential for ferroptosis in all cell types or whether it represents a context-specific mechanism predominant in certain tissues. Second, ALOX15 possesses intrinsic capacity to oxygenate membrane phospholipids independently of PEBP1 [31]; the contribution of this PEBP1-independent activity to ferroptosis signaling has not been systematically evaluated. Third, while PEBP1 knockout mice have been generated and characterized in other contexts, their ferroptosis sensitivity has not been specifically examined, representing an important avenue for future investigation.

ALOX12 (12-Lipoxygenase)

ALOX12 has been linked to ferroptosis through a distinct pathway that is independent of the canonical SLC7A11/GPX4 axis. Chu et al. demonstrated that ALOX12 is required for p53-mediated tumor suppression, through a p53-dependent ferroptosis pathway in which p53 represses SLC7A11, releasing ALOX12 from inhibition and promoting ALOX12-dependent ferroptotic cell death [54]. ALOX12 activation has also been implicated in ferroptosis during toxicant-induced injury.

Questions that remain unresolved include (i) whether Alox12 knockout mice are protected from ferroptosis in in vivo disease models—to date, the evidence is limited to cell culture and xenograft studies; (ii) how ALOX12 substrate specificity (preference for free arachidonic acid rather than esterified phospholipids) shapes its ferroptosis-relevant product profile; and (iii) whether ALOX12 can functionally compensate for ALOX15 loss in tissues where both isoforms are expressed.

ALOX5 (5-Lipoxygenase)

The role of ALOX5 in ferroptosis remains the most controversial among the ALOX isoforms. Unlike ALOX15 and ALOX12, which have been directly implicated in ferroptosis execution, the evidence for ALOX5 involvement is indirect and context-dependent. Some studies have reported that pharmacological inhibition of ALOX5 can modulate ferroptosis sensitivity in specific cell types, but these findings have not been consistently replicated. In Huntington’s disease models, ALOX5-mediated ferroptosis acts as a distinct cell death pathway upon oxidative stress, and Alox5 inactivation is protective [56].

A fundamental question is whether ALOX5, which preferentially oxygenates free arachidonic acid and is primarily localized to the nuclear envelope and cytosol, has sufficient access to membrane phospholipid substrates to generate ferroptotic signals. It is plausible that ALOX5 contributes to ferroptosis indirectly, for example, through the generation of leukotrienes that amplify inflammatory signaling and secondary oxidative stress, rather than through direct phospholipid peroxidation. Whether ALOX5 acts directly on membrane phospholipid substrates or indirectly through leukotriene signaling remains to be resolved in further models.

ALOX15B and Other ALOX Isoforms

ALOX15B, the second mammalian 15-lipoxygenating enzyme, has received considerably less attention in the ferroptosis field than ALOX15. Although ALOX15B shares substrate specificity with ALOX15 for arachidonic acid 15-lipoxygenation, its ability to oxygenate complex esterified substrates and its role in ferroptosis signaling remain poorly characterized. Similarly, ALOX12B and ALOXE3, which are primarily expressed in skin, have not been systematically evaluated for ferroptosis relevance.

The generation and characterization of compound knockout mice (e.g., Alox15-/- plus Alox5-/-, or Alox15-/- plus Alox12-/-) would be particularly valuable for assessing functional redundancy among ALOX isoforms in ferroptosis susceptibility. Such studies would help determine whether individual ALOX isoforms serve non-redundant functions in ferroptosis execution or whether their combined activity is required.

In summary, while substantial evidence implicates ALOX15 and ALOX12 in ferroptosis signaling through distinct mechanisms, systematic comparisons across isoforms, tissues, and in vivo models remain lacking (Table 2 summarizes the current evidence and knowledge gaps for each ALOX isoform.). The field would benefit greatly from standardized ferroptosis assays applied to cells derived from single and compound ALOX knockout mice, which would establish the relative contribution of each isoform and reveal potential redundancies. Until such data are available, conclusions about the specific roles of individual ALOX isoforms in ferroptosis must be drawn with appropriate caution.

### 4.6. Protective LOX Actions

The lipid barrier model suggests a more nuanced relationship between lipid oxygenation and ferroptosis than is typically appreciated. While abundant evidence implicates lipid peroxidation as the executioner mechanism of ferroptosis, not all lipid oxygenation is ferroptotic, and some may be distinctly protective.

Lipoxin A_4_ (LXA_4_), a product of sequential LOX action (ALOX5 and ALOX15) on arachidonic acid, provides the most direct evidence that enzymatic lipid oxygenation can be anti-ferroptotic. Kollareth et al. (2025) demonstrated that LXA_4_ protects alveolar epithelial cells from ferroptosis during lung ischemia–reperfusion injury [10]. LXA_4_ signaling through its receptor FPR2 activated the NRF2 pathway, leading to enhanced expression of antioxidant enzymes and suppression of lipid peroxidation. This study is particularly significant because it demonstrates that a specific SPM, the product of sequential LOX actions, can attenuate ferroptosis in a disease-relevant model [10] (Figure 2, upper pathway).

NRF2 as the Convergence Point: The NRF2 pathway emerges as a critical convergence node linking protective lipid oxygenation to ferroptosis resistance. NRF2 transcriptional targets include not only classic antioxidant enzymes (HO-1, NQO1) but also proteins directly relevant to ferroptosis regulation, including SLC7A11 (the substrate-specific subunit of system Xc^−^) and enzymes involved in glutathione and NADPH synthesis. Electrophilic oxylipins that activate NRF2 demonstrated by Kansanen et al. [5] and Zheng et al. [6] may therefore confer ferroptosis resistance through multiple parallel mechanisms.

Protective evidence is no longer confined to a single study, organ or mediator: RvD1/FPR2 signaling mitigates ferroptosis of macrophages in experimental abdominal aortic aneurysm [62]; maresin-1 alleviates lipid peroxidation-induced ferroptosis after brain injury [63]; and LXA4 also protects erastin-treated primary spinal cord neurons through Akt/NRF2/HO-1 signaling [64]. The shared signaling mechanisms (GPCR engagement, NRF2 activation, pro-resolving transcriptional programs) suggest that resolvins, protectins, and maresins act similarly, although each has so far been demonstrated in a limited set of models (Table 3).

### 4.7. Ferroptosis Resistance States

The concept of acquired ferroptosis resistance has emerged as a critical area of investigation, particularly in cancer therapy. Viswanathan et al. (2017) identified that therapy-resistant, mesenchymal-state cancer cells are exquisitely dependent on GPX4 for survival, suggesting that these cells occupy a “high-lipid-peroxidation” state requiring active suppression to prevent ferroptosis [65].

This state of acquired ferroptosis resistance, which may be understood as a form of “ferroptosis adaptation”, can be interpreted through the lipid barrier framework as a state in which the barrier has been actively reinforced. In this model, cells in a ferroptosis-resistant state maintain an active barrier through: (1) enhanced LOX activity that channels PUFA metabolism toward protective oxylipins rather than destructive peroxidation; (2) constitutive NRF2 activation that drives GPX4 and system Xc^−^ expression; and (3) SPM autocrine signaling that sustains the pro-resolving, anti-ferroptotic phenotype. This framework predicts that interventions which disrupt the lipid barrier, for example, by LOX inhibition or NRF2 antagonism, could sensitize resistant cells to ferroptosis induction, a strategy of potential therapeutic value.

## 5. Therapeutic Opportunities and Future Directions

### 5.1. Enhancing the Lipid Barrier: Therapeutic Opportunities in Ferroptosis-Associated Pathologies

Enhancing the barrier can be approached in two principal ways (Table 4, upper row). First, direct supplementation with stable SPM analogs or oxylipin mimetics, an approach already under clinical investigation for inflammatory conditions, could bypass the need to modulate enzyme activity. The success of LXA_4_ in suppressing ferroptosis in the lung ischemia–reperfusion model [10] provides a proof-of-concept for this strategy. Second, selective pharmacological activation of specific LOX isoforms (e.g., ALOX15) in target tissues could enhance endogenous barrier formation, though this approach requires careful isoform selectivity to avoid off-target pro-inflammatory effects that might arise from non-specific LOX activation. Currently available LOX-modulating agents lack sufficient isoform selectivity for precise therapeutic targeting, and non-specific LOX activation carries the risk of unintended pro-inflammatory effects.

### 5.2. Disrupting the Lipid Barrier: Sensitizing Cancer Cells to Ferroptosis

In cancer contexts where ferroptosis resistance limits therapeutic efficacy, strategies to overcome the lipid barrier could be valuable (Table 4, lower row). The evidence available does not, however, support combining LOX inhibitors with ferroptosis inducers. Most clinical LOX inhibitors are radical-trapping antioxidants, so their effects cannot be attributed to LOX inhibition [12]; and the selective 15LOX/PEBP1-complex inhibitors suppress ferroptosis rather than sensitizing cells to it [52]. Testing whether LOX activity contributes to ferroptosis resistance therefore requires genetic or isoform-selective elimination of LOX function rather than pharmacological pan-LOX inhibition, and no pharmacological LOX-inhibition strategy is proposed here.

### 5.3. Future Directions and Outstanding Questions

Several key questions remain unanswered regarding the lipid barrier model, its regulation, and its translational potential.

First, the field would benefit from quantitative tools to assess the balance between directed and non-enzymatic lipid oxygenation in biological samples. We have proposed a Lipid Oxygenation Index (LOI), defined as the ratio of stereospecific enzyme-derived oxylipins to racemic free radical-derived peroxidation products, as a conceptual research tool for this purpose [21]. Two clarifications are given here. First, how the index is calculated: oxidized lipid species are resolved by chiral LC-MS/MS and each species is assigned an enantiomeric excess (ee) and a regioisomer profile. Species with high ee and a single dominant regioisomer are classified as enzymatically derived, whereas racemic species containing multiple regioisomers are classified as non-enzymatically derived. The index is then LOI = sum (enzymatic species, pmol per mg lipid)/sum (non-enzymatic species, pmol per mg lipid), and it is interpreted as follows: LOI > 1 indicates that directed oxygenation predominates, LOI < 1 indicates autoxidation, and a value near 1 indicates a mixed regime. Second, how the index should not be used: it is a research tool, not a clinically validated biomarker. We wish to emphasize that LOI is not proposed as a clinically realistic biomarker. Considerable practical limitations remain unresolved, including analytical reproducibility, biological variability, tissue specificity, temporal changes during disease progression, dietary influences, and sampling conditions. Technically, enantiomer-specific internal standards are required; several oxylipin species are unstable during sample processing; and distinguishing enzyme-derived from non-enzyme-derived products that yield the same positional isomer—although enantiomeric excess provides a strong diagnostic signature—remains demanding. At present, LOI remains a conceptual framework for thinking about barrier integrity rather than a validated analytical tool; any application to patient stratification would require substantial analytical and clinical validation.

Beyond methodological development, fundamental biological questions persist:

1. LOX specificity and regulation: Which specific LOX isoforms contribute to the lipid barrier in different tissues, and how is their activity regulated by cellular context, substrate availability, and post-translational modifications?
2. Threshold effects: What determines the transition from protective enzymatic lipid oxygenation to destructive non-enzymatic peroxidation? Is there a quantifiable threshold of oxidative challenge beyond which the barrier is overwhelmed?
3. Redundancy and compensation: To what extent can different LOX isoforms compensate for one another in maintaining the lipid barrier? For example, does ALOX15-1 deficiency lead to compensatory upregulation of ALOX12 or ALOX5, and does this compensation maintain the lipid barrier or qualitatively alter it?
4. Tissue specificity: Does the lipid barrier operate uniformly across tissues, or are there tissue-specific specializations? For example, how does the high PUFA content and oxygen consumption of neural tissue influence the barrier?
5. Therapeutic window: Can the barrier be therapeutically modulated without perturbing essential physiological functions of LOXs and their products?
6. Evolutionary conservation: How conserved is the lipid barrier concept across species, and what can evolutionary studies tell us about its ancestral function?

### 5.4. Testable Predictions of the Lipid Barrier Model

If the lipid barrier model is correct, it must generate predictions that can be falsified experimentally. We propose four such predictions.

Prediction 1: Cells with high ALOX15/GPX4 co-expression should be more resistant to ferroptosis than cells expressing either enzyme alone, because barrier function requires both the generation (LOX) and the detoxification (GPX4) of hydroperoxides. This can be tested by comparing isogenic cell lines with graded ALOX15 and GPX4 expression in erastin- and RSL3-induced ferroptosis assays.

Prediction 2: The Lipid Oxygenation Index (LOI)—the ratio of stereospecific to racemic lipid oxidation products—should correlate with ferroptosis resistance across cell types. Lines with high enantiomeric excess of LOX products (high LOI) should resist ferroptosis induction better than lines dominated by racemic autoxidation products.

Prediction 3: Targeted disruption of SPM signaling, for example, genetic ablation of FPR2, should increase ferroptosis sensitivity in tissues where LXA4/FPR2/NRF2 signaling has been documented [10], and should have no effect where FPR2 is expressed at low or undetectable levels (for example the central nervous system parenchyma and mesenchymal compartments); a positive result in the latter would falsify the model.

Prediction 4: Pharmacological enhancement of GPX4 (e.g., through selenium supplementation) should synergize with LOX activation to confer ferroptosis resistance beyond either intervention alone.

We emphasize that these predictions are currently untested; they are intended to define the experimental agenda that would validate or refute the lipid barrier hypothesis.

## 6. Conclusions

The evidence reviewed here establishes that enzymatically generated lipid oxygenation products can, under defined conditions, oppose uncontrolled lipid peroxidation and ferroptosis. Three findings are experimentally established. First, LOX catalysis and peroxide-reducing enzymes are functionally coupled: GPX4 reduces LOX-derived hydroperoxides and preserves the stereospecificity of LOX oxygenation [4,17]. Second, defined LOX products—including 15-HETE, 13-HODE, and the SPM lipoxin A4—activate cytoprotective programs through PPARs and the NRF2 pathway [5,6,7], and LXA4/FPR2 signaling has been shown to suppress ferroptosis of alveolar epithelial cells in vivo [10]. Third, LOX enzymes can also promote ferroptosis, as exemplified by ALOX15/PEBP1-mediated PE oxidation [50] and ALOX15-dependent myocardial ischemia–reperfusion injury [11].

What remains hypothetical is the claim that these individual observations reflect a unified “lipid antioxidant barrier”—a regulated zone of controlled enzymatic oxygenation that systemically opposes chain peroxidation. Substrate sequestration as a quantitatively significant mechanism, the generalizability of protective oxylipin signaling beyond LXA4, and the evolutionary priority of antioxidant over signaling functions all remain working hypotheses.

The take-home messages of this review are the following. Enzymatic lipid oxygenation and non-enzymatic lipid peroxidation are mechanistically distinct: only the latter propagates as a self-sustaining chain reaction, so the mode of oxidation rather than the amount of oxidized lipid determines whether oxidation is contained. LOX catalytic activity is not protective per se: it contributes to resistance only when the coupled reduction machinery (GPX4, GSH, CoQ/FSP1) is sufficient and substrate access is not redirected to phospholipids, and it contributes to ferroptotic execution when reduction fails. The strongest current support for a protective arm is product- and receptor-mediated, and it remains pharmacological rather than a demonstration of an endogenous barrier. Substrate sequestration is quantitatively insufficient in mammalian membranes and is no longer part of the model’s core. The value of the framework lies in its falsifiable predictions (Section 5.4), not in the claim of an established barrier. The model generates specific, falsifiable predictions (Section 5.4): co-expression of ALOX15 and GPX4 should confer ferroptosis resistance beyond either enzyme alone; the ratio of stereospecific to racemic oxidation products should track ferroptosis resistance; disruption of SPM signaling (e.g., FPR2 knockout) should increase ferroptosis sensitivity; and pharmacological GPX4 enhancement should synergize with LOX activation. Testing these predictions in defined ferroptosis models—erastin- or RSL3-induced ferroptosis, ischemia–reperfusion injury, and GPX4-deficient systems—will determine whether the lipid antioxidant barrier represents a genuine biological principle or a convenient narrative.

## Figures and Tables

**Figure 1:**
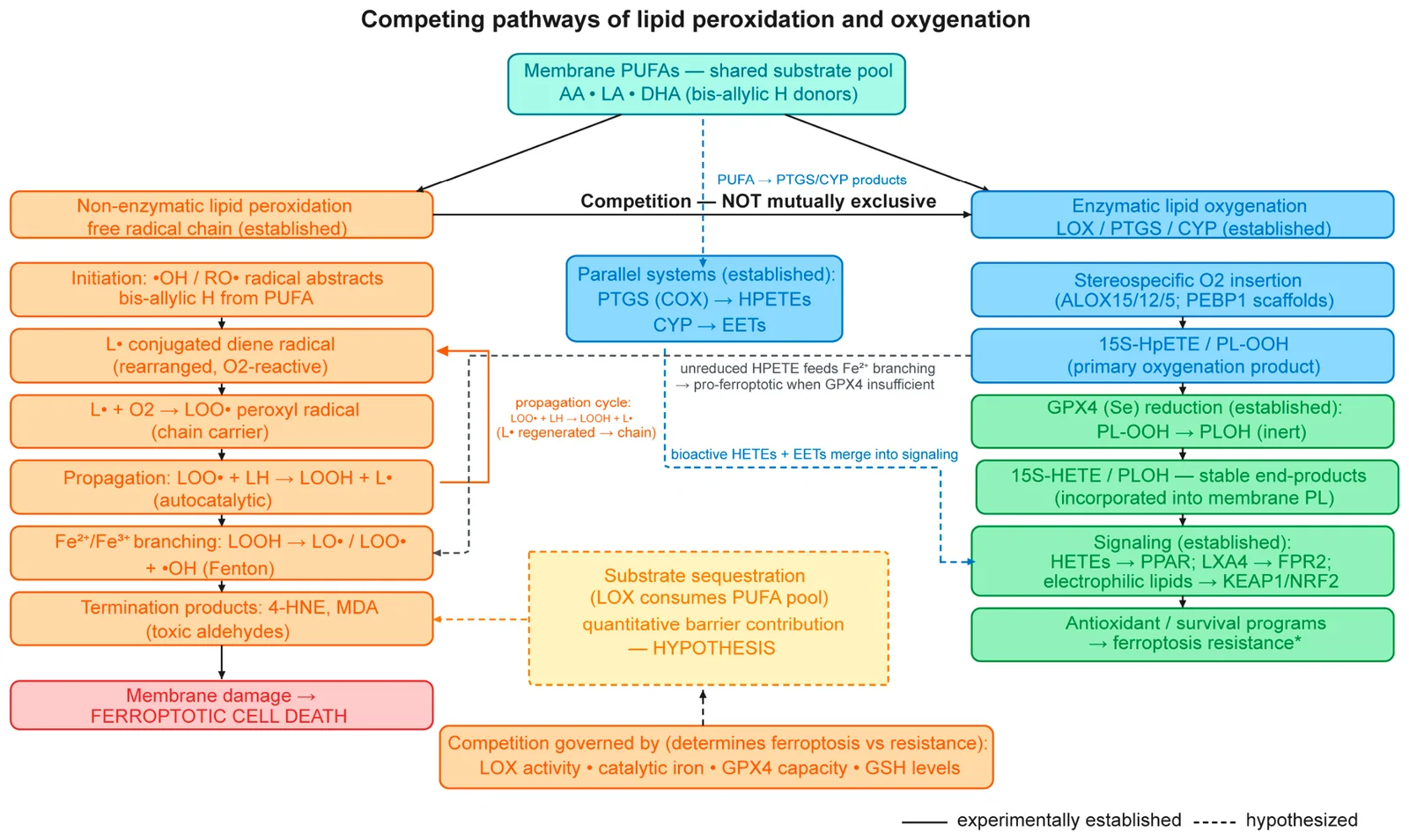
Competing (non-mutually exclusive) pathways of lipid peroxidation and oxygenation. Membrane polyunsaturated fatty acids (PUFAs; arachidonic acid, linoleic acid, and docosahexaenoic acid) constitute a shared substrate pool that feeds two competing—but not mutually exclusive—pathways of lipid oxidation. (**Left**) Non-enzymatic lipid peroxidation proceeds through an iron (Fe^2+^)-catalyzed free radical chain reaction: initiation by •OH/RO• abstraction of a bis-allylic hydrogen; propagation via L• → LOO• (chain cycle: LOO• + LH → LOOH + L•, regenerating L•); Fe^2+^/Fe^3+^-dependent branching of LOOH; and termination yielding reactive aldehydes (4-HNE, MDA) and other toxic products that culminate in membrane damage and ferroptotic cell death. (**Right**) Directed enzymatic lipid oxygenation by lipoxygenases (LOXs; ALOX15/12/5, with PEBP1 scaffolds), together with parallel cyclooxygenase (PTGS) and cytochrome P450 (CYP) systems, generates HPETEs and EETs. HPETEs are reduced by GPX4 to stable HETEs, which—together with specialized pro-resolving mediators (SPMs, e.g., LXA4)—activate protective signaling through PPAR and FPR2; NRF2 activation is driven principally by electrophilic lipids such as nitro-fatty acids. The balance between these pathways is governed by LOX activity, catalytic iron availability, GPX4 capacity, and glutathione levels. Dashed elements denote hypothesized components: substrate sequestration (its quantitative contribution to barrier function) and reflux of unreduced HPETE into Fe^2+^-dependent branching, which becomes pro-ferroptotic when GPX4 is insufficient. Note that a basal level of non-enzymatic peroxidation is required for redox homeostasis, and that uncontrolled enzymatic oxygenation can itself become deleterious; the outcome of each pathway is therefore context-dependent.

**Figure 2:**
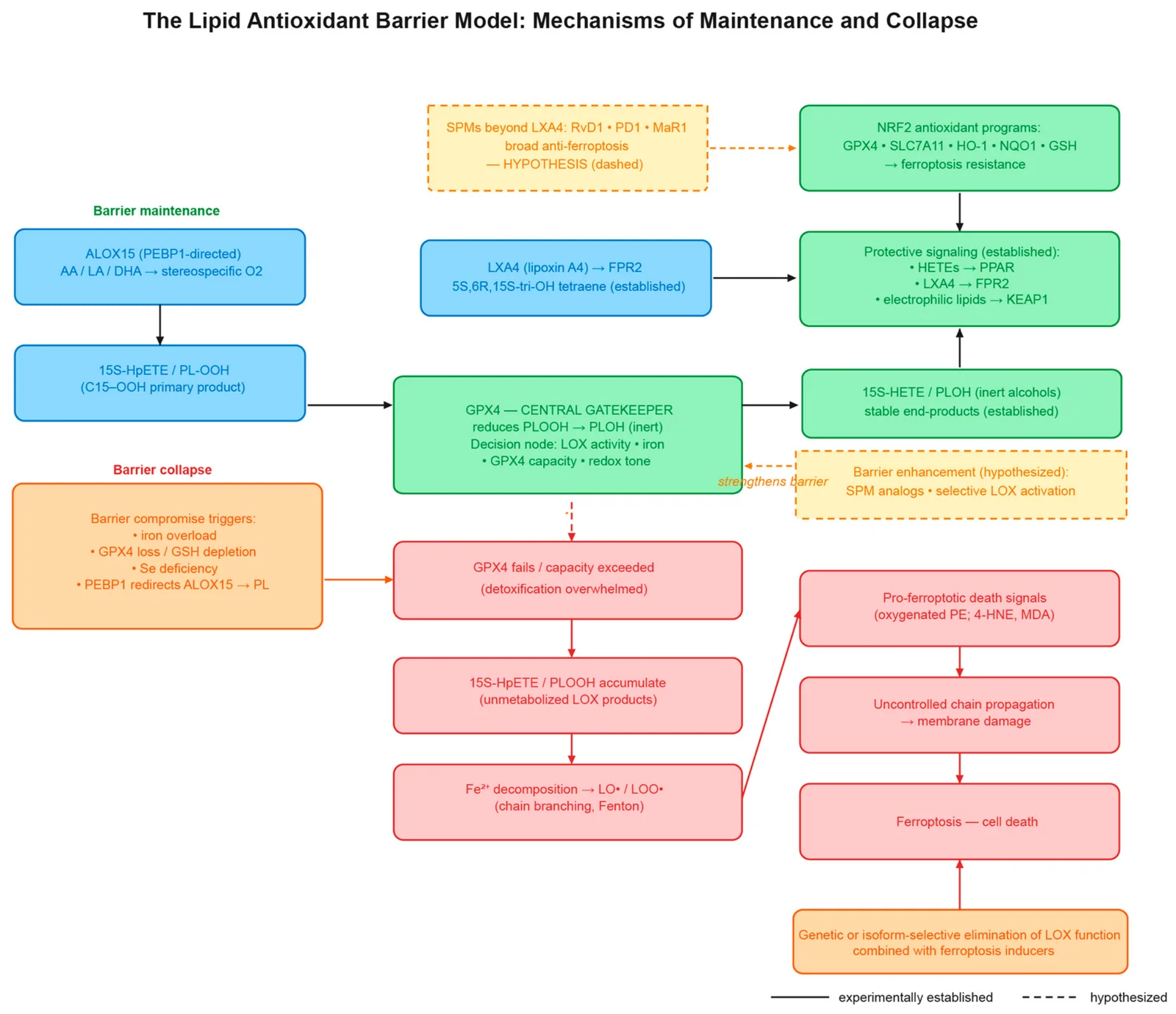
The Lipid Antioxidant Barrier Model: Mechanisms of Maintenance and Collapse. The lipid antioxidant barrier is depicted as a dual-state system with GPX4 as its central gatekeeper. Solid arrows denote experimentally established relationships; dashed arrows denote hypothesized ones. Barrier maintenance (upper pathway). ALOX15 (PEBP1-directed) stereospecifically oxygenates membrane PUFAs (AA/LA/DHA) to 15S-HpETE/PL-OOH. GPX4 (selenium-dependent) reduces PL-OOH to inert PLOH alcohols, preventing unmetabolized hydroperoxides from entering Fe^2+^-dependent decomposition. Stable HETE/PLOH end-products feed protective signaling via PPAR, FPR2 (LXA4) and KEAP1/NRF2, and NRF2-driven programs (GPX4, SLC7A11, HO-1, NQO1, glutathione synthesis) sustain a state of heightened ferroptosis resistance. LXA4, a product of sequential ALOX5 and ALOX15 action, provides the most direct evidence that enzymatic oxygenation can be anti-ferroptotic: LXA4–FPR2 signaling activates NRF2 and suppresses lipid peroxidation. The hypothesis that SPMs beyond LXA4 (RvD1, PD1, MaR1) exert broad anti-ferroptotic activity is shown as a dashed branch feeding the NRF2 program. Barrier collapse (lower pathway). Barrier compromise—iron overload, GPX4 loss or GSH depletion, selenium deficiency, or PEBP1 redirecting ALOX15 toward phospholipid substrates—causes GPX4 to fail or become overwhelmed. Unmetabolized 15S-HpETE/PLOOH accumulate and undergo Fe^2+^-dependent decomposition (Fenton-type branching), generating pro-ferroptotic death signals (oxygenated phospholipids, 4-HNE, MDA) that drive uncontrolled chain propagation, membrane damage and ferroptotic cell death. Thus, the same LOX enzymes that maintain the barrier fuel ferroptosis once the barrier collapses. The GPX4 box is the decision node integrating LOX activity, catalytic iron availability, GPX4 capacity and cellular redox tone. Therapeutic nodes are indicated at the bottom: barrier enhancement (SPM analogs, selective LOX activation; hypothesized) versus barrier disruption (LOX inhibitors plus ferroptosis inducers; in testing).

**Table 1:** Chemical classification of oxylipins and their signaling mechanisms.

Chemical Class	Representative Molecules	Signaling Mechanism	Functional Outcome
Hydroperoxides (free and phospholipid-esterified)	15S-HpETE, 12S-HpETE	No direct receptor activity; metabolic intermediates	Detoxified by GPX4 to HETEs (protective); if unreduced, iron-dependent decomposition promotes ferroptosis
Monohydroxy derivatives	15-HETE, 13-HODE	PPARgamma/alpha; incorporation into membrane phospholipids	Metabolic signaling and membrane remodeling; stable end-products
Electrophilic lipids	Nitro-fatty acids, cyclopentenone prostaglandins	KEAP1 cysteine modification → NRF2	Antioxidant gene induction (HO-1, NQO1); partly non-LOX origin
Specialized pro-resolving mediators	LXA4, resolvin D1, protectins, maresins	GPCRs (FPR2/ALX, GPR32)	Resolution of inflammation; LXA4/FPR2/NRF2 anti-ferroptotic signaling [10]
Leukotrienes (ALOX5 products)	LTB4, cysteinyl-leukotrienes	BLT/CysLT GPCRs	Pro-inflammatory (context-dependent); ferroptosis relevance unresolved

**Table 2:** Summary of Evidence and Knowledge Gaps.

ALOX Isoform	Pro-Ferroptotic Evidence	In Vivo KO Data	Key Knowledge Gaps
ALOX15	PEBP1-mediated PE oxidation; selective inhibitors identified	Alox15-/- mice have been reported; ferroptosis sensitivity not yet systematically tested in vivo;	PEBP1-independent contribution? Tissue-specific requirement?
ALOX12	p53-dependent ferroptosis pathway executed through ALOX12 (p53 represses SLC7A11, releasing ALOX12); independent of GPX4-mediated reduction	Alox12-/- mice available; limited in vivo ferroptosis data	Substrate access? Redundancy with ALOX15?
ALOX5	Indirect; pharmacological data confounded by the radical-trapping activity of the inhibitors	Alox5-/- mice available; ALOX5-dependent ferroptosis reported in Huntington’s disease models	Direct vs. indirect mechanism? Leukotriene involvement?
ALOX15B	Minimal characterization in ferroptosis context	Alox15b-/- mice available	Substrate specificity? Tissue distribution?
ALOX12B/ALOXE3	Not evaluated	Knockout mice exist (skin phenotype)	Skin-specific ferroptosis relevance?

**Table 3:** Documented evidence for protective and counter-protective LOX-mediated lipid oxygenation.

Model System/Study	LOX Isoform or Oxylipin Product	Protective or Pro-Ferroptotic Mechanism	Strength of Evidence
Mouse lung ischemia–reperfusion injury; alveolar epithelial cells [10]	LXA4 (sequential ALOX5/ALOX15 product) via FPR2/ALX	NRF2 activation, increased GSH, decreased MDA; ferroptosis suppression	Strong (in vivo with genetic support: protection abolished in Fpr2-/- and Nrf2-/- mice); one study in this organ, with support from other mediators and organs (Section 4.6)
In vitro, 15-LOX with complex substrates [4]	ALOX15 HPETEs	GPX4-coupled immediate reduction; stereospecificity of oxygenation preserved	Mechanistic (in vitro); supports the coupled LOX/GPX4 module
Cell-based NRF2 studies [5,6]	Nitro-fatty acids (lipid oxidation products; not LOX-derived)	KEAP1 cysteine modification → NRF2 activation; HO-1, heat shock proteins, thioredoxin reductase induction	Established for NRF2 activation by lipid oxidation products; does not establish LOX products per se
In vitro transactivation [7]	15-HETE, 13-HODE	Endogenous PPARgamma/alpha ligands	Established (in vitro)
Resolution physiology [8,9]	SPMs (lipoxins, resolvins, protectins, maresins)	GPCR engagement, pro-resolving transcriptional programs	Established for inflammation resolution; anti-ferroptotic evidence limited to LXA4
Cell and mouse models of ferroptosis [59,60]	ALOX15 (12/15-LOX)	Counter-evidence: LOX-dependent ferroptosis; 12/15-LOX-dependent death upon GPX4 loss	Strong (pharmacological and genetic)
Biochemical, cellular and structural studies [50,61]	ALOX15/PEBP1 complex	Counter-evidence: oxygenated PE species as ferroptotic death signals	Strong (biochemistry, cells, structure)

**Table 4:** Therapeutic Strategies Targeting the Lipid Antioxidant Barrier.

Strategy	Approach	Therapeutic Context	Key Consideration
Enhancing the Barrier	SPM analogs (e.g., stable LXA4 mimetics); selective inhibition of the 15LOX/PEBP1 complex, which suppresses ferroptosis without interfering with ALOX15 biosynthesis.	Ferroptosis-associated pathologies (ischemia–reperfusion injury, neurodegeneration)	Requires isoform selectivity to avoid non-specific pro-inflammatory LOX activation [11,52]
Disrupting the Barrier	Ferroptosis inducers (e.g., erastin, RSL3) combined with genetic or isoform-selective elimination of LOX function. Non-selective pharmacological LOX inhibitors are not suitable for this purpose, because their cytoprotective effects are attributable to radical-trapping antioxidant activity rather than to enzyme inhibition.	Cancer therapy (sensitizing mesenchymal/chemoresistant tumors)	Must be tumor-targeted to prevent systemic barrier collapse and ferroptosis in healthy tissues [24,65]

## Data Availability

No new data were created or analyzed in this study. Data sharing is not applicable to this article.

## Abbreviations

AA, arachidonic acid; ACSL4, acyl-CoA synthetase long-chain family member 4; ALOX, arachidonate lipoxygenase; BH4, tetrahydrobiopterin; CYP, cytochrome P450; DHA, docosahexaenoic acid; DHODH, dihydroorotate dehydrogenase; EPA, eicosapentaenoic acid; FPR2/ALX, formyl peptide receptor 2/lipoxin A4 receptor; FSP1, ferroptosis suppressor protein 1; GCH1, GTP cyclohydrolase 1; GPX4, glutathione peroxidase 4; GSH, glutathione; HETE, hydroxyeicosatetraenoic acid; HODE, hydroxyoctadecadienoic acid; HO-1, heme oxygenase-1; HPETE, hydroperoxyeicosatetraenoic acid; KEAP1, Kelch-like ECH-associated protein 1; LA, linoleic acid; LPCAT3, lysophosphatidylcholine acyltransferase 3; LOI, lipid oxygenation index; LOX, lipoxygenase; LXA4, lipoxin A4; MDA, malondialdehyde; NQO1, NAD(P)H:quinone oxidoreductase 1; NRF2, nuclear factor erythroid 2-related factor 2; PE, phosphatidylethanolamine; PEBP1, phosphatidylethanolamine-binding protein 1; PPAR, peroxisome proliferator-activated receptor; PTGS, prostaglandin–endoperoxide synthase; PUFA, polyunsaturated fatty acid; SPM, specialized pro-resolving mediator; 4-HNE, 4-hydroxynonenal.
